# Receipt of Behavioral Health Services Among US Children and Youth With Adverse Childhood Experiences or Mental Health Symptoms

**DOI:** 10.1001/jamanetworkopen.2021.1435

**Published:** 2021-03-15

**Authors:** David Finkelhor, Heather Turner, Deirdre LaSelva

**Affiliations:** 1 Crimes Against Children Research Center, University of New Hampshire, Durham; 2 Department of Sociology, University of New Hampshire, Durham

## Abstract

**Importance:**

Epidemiological literature on children’s mental health and children’s adverse childhood experiences (ACEs) have consistently pointed to widespread, unaddressed, and treatable high-risk conditions among children.

**Objective:**

To estimate the proportion of children with either high levels of ACEs and/or high levels of mental health symptoms who were not receiving services from behavioral health professionals.

**Design, Setting, and Participants:**

This cross-sectional study included 11 896 children who participated in 3 National Surveys of Children’s Exposure to Violence (NatSCEV), which were nationally representative surveys conducted in 2008, 2011, and 2014. The surveys entailed telephone interviews with youth aged 10 to 17 years and caregivers of children aged 2 to 9 years. Data were analyzed from February to August 2020.

**Main Outcomes and Measures:**

Nationally representative samples were obtained from a mix of random digit dial and address-based sampling methods. The primary outcome was the proportion of children with high ACEs, high distress symptoms, and both who were receiving clinical contact, broken down by demographic characteristics.

**Results:**

Of the 11 896 children, 4045 (34.0%) participated in the 2008 NatSCEV; 4112 (34.6%) in the 2011 NatSCEV; and 3738 (31.4%) in the 2014 NatSCEV; 5532 (46.5%) were aged 2 to 9 years (2785 [50.4%] aged 2-5 years; 2693 [48.7%] girls; 3521 [63.7%] White children), and 6365 (53.5%) were aged 10 to 17 years (3612 [56.7%] aged 14-17 years; 3117 [49.0%] female participants; 4297 [67.5%] White individuals). Among participants aged 2 to 9 years, no clinical contact was reported for 57% (95% CI, 51%-62%) of the high ACE group, 53% (95% CI, 48%-58%) of the high distress symptom group, and 41% (95% CI, 32%-51%) of the group with high levels on both indicators. Among participants aged 10 to 17 years, the group with no clinical contact comprised 63% (95% CI, 56%-69%) of the high ACE group, 52% (95% CI, 46%-57%) of the high distress symptom group, and 62% (95% CI, 51%-71%) of youth scoring high on both indicators. Among racial groups, odds of contact were very low for Black children aged 2 to 9 years with high ACEs compared with non-Hispanic White children with the same age and exposure (odds ratio, 0.26; 95% CI, 0.14-0.49).

**Conclusions and Relevance:**

In this cross-sectional study combining findings from 3 US national surveys, large portions of children at high risk because of adversity or mental health symptoms were not receiving clinical services. Better ways are needed to find these at-risk populations and help them obtain relevant intervention resources.

## Key Points

**Question:**

What proportion of children and youth who have distress symptoms and/or adverse childhood experiences (ACEs), which often lead to later behavioral and physical health problems, do not receive behavioral health services?

**Findings:**

In this cross-sectional study of 11 896 children and youth, no current clinical behavioral health contact was reported for 57% of children aged 2 to 9 years with high ACEs, 53% of children aged 2 to 9 years with high distress symptoms, and 41% of children aged 2 to 9 years with high levels on both indicators. Among youth aged 10 to 17 years, no current clinical contact was reported for 63% of those with high ACEs, 52% of those with high distress symptoms, and 62% of those who scored high on both.

**Meaning:**

These findings suggest that large portions of the high-risk youth population are not receiving behavioral health services that could improve their developmental outcomes.

## Teaser

This cross-sectional study estimates the proportion of children with high levels of adverse childhood experiences (ACEs) and/or high levels of mental health symptoms who were not receiving services from behavioral health professionals.

## Introduction

Two lines of research have consistently pointed to deficiencies in efforts to find and help high-risk children and youth. One is from the literature that focuses on undiagnosed and untreated mental health and behavioral problems.^1^ The other is from the literature on adverse childhood experiences (ACEs),^2^ life exposures that have been found to be strongly associated with both early and later emotional and behavioral problems. Both literatures have argued for the need to make a greater effort to identify such problems in children, possibly by developing protocols for universal screening. However, debates exist about how to go about such screening, who to screen, and what to screen for.

The literature concerned with mental health screening highlights epidemiologic studies showing that as much as one-quarter of the child and youth population experiences mental health disorders,^3^ but only one-third to one-half of those with disorders get treatment.^3,4,5,6^ Advocates point out that effective treatments exist for many of these conditions and have urged universal screening, especially for widespread, specific, and treatable problems, such as anxiety^1^ and depression.^7^ Universal screening experiments to identify mental health symptoms have been fielded in some locales.^8^

For its part, the ACE literature has identified a group of childhood experiences that have been shown to be widespread in the population—including physical and sexual abuse, parental drug and alcohol abuse, and interpersonal violence^9^—that are also associated with a high risk of physical and mental health problems later in life.^10,11,12^ An accumulation of these adversities has been also found to be strongly associated with mental health problems in childhood and adolescence.^13^ Advocates have argued that children with these multiple adversities need to be screened and flagged for intervention, much if not all of it through referral to behavioral health practitioners. Programs for universal screening in pediatric care have been rolled out statewide in California^14^ and in some large community health systems.^15^

There are many issues raised by these 2 somewhat different approaches to risk assessment and intervention for youth. One important question is the degree to which such high-risk groups overlap. Another is which approach better identifies the youth most likely to benefit from services. Another is how many of the children identified by each of these risk assessment strategies are already currently engaged with behavioral health services. High rates of engagement with the service system among high-risk youth would be reassuring. We might also expect that children showing distress symptoms would be more visible and, as a result, more likely to enter treatment than children who simply have adversity risk factors, at least at some ages. It also is important to identify segments of the population for which detection and treatment seem to be most effective and which are most underserved.

This study uses information from the aggregation of 3 national samples of children and youth to assess how likely it is that behavioral health services have been received among children with elevated mental health symptoms, ACEs, or both. It will also examine demographic features that are associated with more service receipt, eg, a possible differential by race/ethnicity, which has been noted in some literature.^16^

## Methods

### Sample and Procedure

The analyses that follow use data from 3 National Surveys of Children’s Exposure to Violence (NatSCEV), cross-sectional US studies that collected information from nationally representative samples of children and youth aged 1 month to 17 years in 2008, 2011, and 2014. All study materials were reviewed and approved by the University of New Hampshire institutional review board. Consent was obtained from parents, and assent was obtained from youth who were interviewed. All data used for analysis were deidentified. This study followed the Strengthening the Reporting of Observational Studies in Epidemiology (STROBE) reporting guideline.

The samples from each of the 3 surveys were obtained from a mix of random digit dialing, address-based sampling, and targeted oversampling of households with children, cell phone–only households, and/or underrepresented racial groups. Using the American Association for Public Opinion Research (AAPOR) response rate 4 calculation, the NatSCEV I survey (2008) achieved a 55% response rate; NatSCEV II (2011), 44.6%; and NatSCEV III (2014), 24.1%.^17^ The decline in response rates between NatSCEV I and III is consistent with the overall national trend in telephone and other survey modes.^18,19^

Interviews began with an adult caregiver in each household to collect family demographic information. One child was randomly selected from all eligible children living in a household by sampling the child with the most recent birthday. Demographic information about child’s age, gender, race, parental education, family structure, and type of location were provided by the caregivers. Race information was collected because of well-established disparities in abuse, crime, and adversities by race. Telephone interviews were conducted with children 10 years and older about their adversity experiences, symptoms, and other topics. If the selected child was younger than 10 years, proxy interviews were conducted by the caregiver “who was most familiar with the everyday experiences of the child.”

Sample weights adjusted for differential probability resulting from both the complex study design as well as variations within household eligibility and nonresponse by demographic characteristics. More information about the sample and weighting is available in prior publications.^20,21,22^ The analysis for the current study used pooled data from all 3 surveys (2008, 4045 participants; 2011, 4112 participants; 2014, 3738 participants) for a total sample size of 11 896 (5532 [46.5%] for aged 2-9 years; 6364 [43.5%] aged 10-17 years).

Interviews averaged approximately 50 minutes in length and were conducted in English or Spanish. Respondents who disclosed a situation of serious threat or ongoing abuse were recontacted by a clinical member of the research team, trained in telephone crisis counseling, whose responsibility was to provide them with contact information for support in their local community.

### Measurement

#### ACEs

The NatSCEV interviews included numerous questions and indexes from which to draw a large pool of lifetime ACEs that fell within several content domains: crime, abuse, or violence; family instability; interpersonal loss; parental psychological disorder; environmental threat; and economic stressors. In a previous analysis, the researchers distilled 40 adversities into a set of 15-item inventories most associated with adverse outcomes for each age group.^13^ Based on a sensitivity and specificity analysis, a high ACES group was demarcated by the presence of 5 or more adversities among children aged 2 to 9 years or 7 or more adversities among those aged 10 to 17 years.

#### Psychological Distress

Distress was measured with the anger or aggression, depression, anxiety, dissociation, and posttraumatic stress scales of the Trauma Symptoms Checklist for Children (TSCC) and the Trauma Symptoms Checklist for Young Children (TSCYC), thus including both internalizing and externalizing symptoms. There were 24 symptom items from the TSCC^23^ designed for youth aged 10 years and older and 26 symptom items from TSCYC completed by parents of children aged 2 to 9 years. Both scales assess children in several general mental health symptom domains, including depression, anxiety, anger, posttraumatic stress, and dissociation, without enumerating any specific trauma exposure. Respondents were asked to indicate how often they (or their child) had experienced each symptom within the last month. The TSCC and TSCYC have demonstrated good test-retest and internal consistency reliability and good concurrent validity in clinical and population-based samples.^23,24^ A summary count measure of all symptoms was constructed for each age group. For some analyses, the researchers also constructed a high symptom indicator to identify cases of potential clinical significance. High symptoms were defined, consistent with previous usage of the measure,^13^ as the top decile of the summary measure for each age group.

Clinical behavioral health contact (referred to as clinical contact) was assessed fairly broadly by answers to 4 questions asked of parents. Together they were intended to capture youth’s contact with professionals who, given their training, should be in a position to either provide treatment or to recognize the need for services and make an appropriate referral. The questions included (1) “Does your child currently receive special services at school? These might include an individualized education plan (IEP), 504 plan, or special education services.” (2) “Does your child currently take any medication associated with an emotional, behavioral, or learning problem?” (3) “Has your child had an evaluation or received any counseling for emotional, behavioral, or developmental problems in the last year?” (4) “Has your child ever been diagnosed by a doctor, therapist or another professional with any of the following: post-traumatic stress disorder (PTSD) or other anxiety disorder; attention deficit disorder or attention-deficit/hyperactivity disorder (ADD, ADHD); oppositional/defiant disorder or conduct disorder (ODD or CD); autism, pervasive developmental disorder (PDD), or Asperger; developmental delay or retardation; depression; learning disorders (dyslexia, reading, math or other learning problem).” Educational services were included because they generally mean that a professional has recognized that a child has cognitive or behavioral challenges that need assessment and remedy. To be consistent with questions 1, 2 and 3, which refer to the current or past year, only diagnoses in question 4 that were received at the child’s current age were counted.

### Statistical Analysis

For high-risk groups demarcated by ACES, symptoms, and both, percentages not receiving services were calculated within the age categories and then compared using χ^2^ test. By age group, logistic regressions were calculated using service receipt as the dependent variable and demographic features as independent variable. Data analysis was conducted in Stata/SE version 16.0 (StataCorp). Statistical significance was set at *P* < .05, and all tests were 2-tailed.

## Results

Of the 11 896 children, 4045 (34.0%) participated in the 2008 NatSCEV; 4112 (34.6%) in the 2011 NatSCEV; and 3738 (31.4%) in the 2014 NatSCEV. Table 1 shows the demographic breakdown for the younger and older children. Among the 5532 children (46.5%) aged 2 to 9 years (2785 [50.4%] aged 2-5 years; 2693 [48.7%] girls; 3521 [63.7%] White children), 33% (95% CI, 31%-35%) had at least 1 indicator of a past year clinical contact. Among the 6365 youth (53.5%) aged 10 to 17 years (3612 [56.7%] aged 14-17 years; 3117 [49.0%] female participants; 4297 [67.5%] White individuals), the percentage was 27% (95% CI, 25%-29%). Broken down by type of contact received, among all children aged 2 to 9 years, 11% (95% CI, 9%-12%) received an evaluation or received counseling for emotional, behavioral, or developmental problems; 9% (95% CI, 8%-11%) received a mental health diagnosis; 4% (95% CI, 3%-5%) received a psychiatric medication; and 26% (95% CI, 24%-28%) received services at school. Among all youth aged 10 to 17 years , 13% (95% CI, 12%-15%) received an evaluation or counseling, 6% (95% CI, 5%-7%) received a diagnosis, 9% (95% CI, 8%-11%) received a psychiatric medication, and 14% (95% CI, 13%-16%) received services at school.

**Table 1.** Descriptive Statistics of Study Variables

Characteristic	Children and youth by age, No. (%)	
	2-9 y	10-17 y
Gender		
Male	2839 (51.3)	3248 (51.0)
Female	2693 (48.7)	3117 (49.0)
Age, y		
2-5	2785 (23.4)	NA
6-9	2747 (23.1)	NA
10-13	NA	2753 (23.1)
14-17	NA	3612 (30.4)
Race		
White	3521 (63.7)	4297 (67.5)
Black	785 (14.2)	842 (13.2)
Hispanic	867 (15.7)	893 (14.0)
Other^a^	342 (6.2)	307 (4.8)
Parent education		
<High school	319 (5.8)	413 (6.5)
High school or some college	1856 (33.6)	1980 (31.1)
College	1860 (33.6)	2208 (34.7)
Graduate school	1497 (27.0)	1764 (27.7)
Family structure		
2 Parent	3944 (71.3)	4179 (65.7)
Parent and stepparent	257 (4.7)	616 (9.7)
Single parent	1069 (19.3)	1296 (30.3)
Other adult	262 (4.7)	274 (4.3)
Geographic location		
Urban	3103 (56.2)	3381 (53.3)
Rural	2418 (43.8)	2964 (46.7)
Parental employment		
Employed full or part time	3504 (63.3)	4424 (69.5)
Unemployed	2028 (36.7)	1941 (30.5)
Clinical contact		
Yes	1769 (33.0)	1691 (26.6)
No	3763 (67.0)	4674 (73.4)
High risk categories, mean (SD)		
ACEs total	4.9 (3.8)	7.8 (5.1)
Symptoms	4.6 (7.0)	8.5 (11.7)

Abbreviations: ACE, adverse childhood experience; NA, not applicable.

^a^ Other racial category includes Alaskan Native, American Indian, Asian, or mixed race.

The portion of the sample with a high ACE scores was 16% (95% CI, 15%-18%; 352 children) of those aged 2 to 9 years and 12% (95% CI, 11%-14%; 270 youth) of those aged 10 to 17 years. The portion with high levels of distress symptoms was 11% (95% CI, 9%-13%; 306 children) of those aged 2 to 9 years and 10% (95% CI, 9%-12%; 270 youth) of those aged 10 to 17 years. Approximately 6% of each age sample had the combination of both high ACE scores and high levels of distress symptoms. Among children aged 2 to 9 years with high ACEs, 66% (95% CI, 59%-71%) did not have high symptoms, and of those with high symptoms, 50% (95% CI, 42%-57%) did not have high ACEs. Among those aged 10 to 17 years, the proportion of children with high ACEs but not high symptoms was 61% (95% CI, 54%-67%) and that with high symptoms but not high ACEs was 54% (95% CI, 46%-61%).

Large fractions of the high-risk ACEs and high symptoms risk populations did not report any indicator of clinical contact. Among the those aged 2 to 9 years, no clinical contact was reported for 57% (95% CI, 51%-62%) of the group with high ACEs, 53% (95% CI, 48%-58%) of the group with high symptoms, and 41% (95% CI, 32%-51%) of the group with high levels on both indicators (Figure). Among those aged 10 to 17-years, the no clinical contact group comprised 63% (95% CI, 56%-69%) of youth with high ACEs, 52% (95% CI, 46%-57%) of those with high distress symptoms, and 62% (95% CI, 51%-71%) of those with both indicators. For those aged 2 to 9 years, the group with combined high ACEs and high symptoms had more clinical contact than the other 2 groups (vs high symptoms alone: χ^2^_1_ = 28.88, *P* < .001; vs high ACEs alone: χ^2^_1_ = 47.45; *P* < .001). Among those aged 10 to 17 years, the high symptoms group had significantly less clinical contact than the high ACE group (χ^2^_1_ = 25.17; *P* < .001) or the group with combined high ACEs and high symptoms (χ^2^_1_ = 20.37; *P* < .001).

**Figure.**
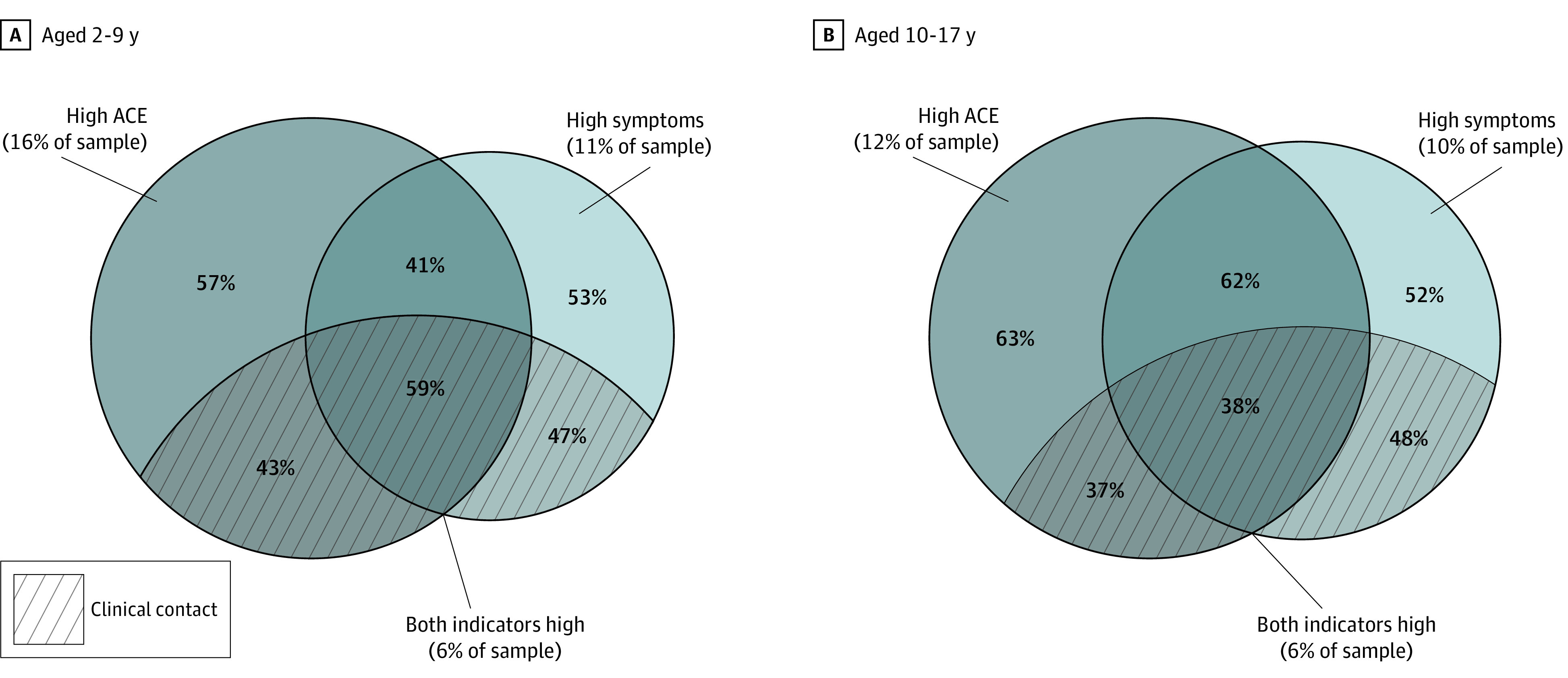
Clinical Contact Among High-Risk Groups

A logistic regression evaluating the likelihood of clinical contact based on demographic factors was performed for each of the 6 age and risk type groups. Clinical contact varied by race and some other demographic variables (Table 2). Controlling for other demographic factors, Black children aged 2 to 9 years with high ACEs and children who identified as another race and had high symptoms were significantly less likely to have clinical contact than non-Hispanic White children their age (Black children: odds ratio, 0.26; 95% CI, 0.14-0.49; other race: 0.43; 95% CI, 0.23-0.83). There were significant disparities for Hispanic youth and youth who identified as other race aged 10 to 17 years compared with non-Hispanic White youth, as well. Relative to those with college-educated parents, younger children who had high ACES and high symptoms with only high school–educated parents were significantly less likely to have clinical contact. Among older children with high symptoms, those whose parents had less than a high school education were also less likely than those with college-educated parents to have contact. Children from family structures with a single parent, stepparents, and other configurations had higher odds of clinical contact for both age groups and both risk measures compared with children in 2-parent households, independent of other demographic factors.

**Table 2.** Adjusted Odds of Clinical Contact Among High-Risk Groups, by Demographic Category

Characteristic	Adjusted odds ratio (95% CI)					
	Children aged 2-9 y			Youth aged 10-17 y		
	High ACE (n = 352)	High symptoms (n = 306)	Both high (n = 192)	High ACE (n = 270)	High symptoms (n = 270)	Both high (n = 138)
Race						
Non-Hispanic White	1 [Reference]	1 [Reference]	1 [Reference]	1 [Reference]	1 [Reference]	1 [Reference]
Black	0.26 (0.14-0.49)^a^	0.55 (0.28-1.05)	0.31 (0.13-0.77)^b^	0.61 (0.24-1.55)	0.64 (0.35-1.16)	0.48 (0.20-1.16)
Hispanic	0.81 (0.42-1.56)	1.16 (0.61-2.20)	1.32 (0.58-3.02)	0.49 (0.25-0.94)^c^	0.67 (0.36-1.25)	0.44 (0.20-1.00)^c^
Other^d^	0.63 (0.31-1.32)	0.43 (0.23-0.83)^b^	0.48 (0.20-1.18)	0.48 (0.21-1.06)	0.38 (0.17-0.85)^c^	0.29 (0.10-0.86)^c^
Gender						
Female	1 [Reference]	1 [Reference]	1 [Reference]	1 [Reference]	1 [Reference]	1 [Reference]
Male	0.72 (0.49-1.06)	0.64 (0.41-0.98)	0.74 (0.41-1.33)	0.96 (0.60-1.55)	1.15 (0.72-1.85)	1.65 (0.91-3.01)
Age as continuous variable	1.05 (0.94-1.18)	0.94 (0.83-1.06)	1.05 (0.90-1.22)	1.18 (1.00-1.35)^b^	1.06 (0.97-1.16)	1.21 (1.06-1.40)^b^
Parental education						
College	1 [Reference]	1 [Reference]	1 [Reference]	1 [Reference]	1 [Reference]	1 [Reference]
<High school	1.58 (0.70-3.55)	1.95 (0.91-4.18)	1.70 (0.62-4.59)	1.57 (0.60-4.13)	5.72 (2.06-15.74)^a^	1.58 (0.48-5.20)
High school or some college	2.07 (1.32-3.25)^a^	1.64 (1.01-2.66)^c^	1.70 (0.89-3.25)	1.00 (0.53-1.90)	1.42 (0.80-2.51)	1.23 (0.54-2.82)
Graduate school	0.88 (0.51-1.54)	1.33 (0.76-2.34)	0.94 (0.44-2.00)	0.92 (0.50-1.67)	0.93 (0.55-1.57)	1.15 (0.56-2.36)
Family structure						
2 Parents	1 [Reference]	1 [Reference]	1 [Reference]	1 [Reference]	1 [Reference]	1 [Reference]
Other adult	9.00 (5.06-16.00)^a^	5.12 (2.85-9.20)^a^	8.12 (4.10-16.07)^a^	2.17 (0.98-4.83)	2.94 (1.21-7.24)^c^	1.60 (0.60-4.34)
Parent and stepparent	2.72 (1.47-5.05)^a^	1.73 (0.85-3.56)	3.00 (1.33-6.63)^a^	4.91 (2.45-9.84)^a^	3.49 (1.71-7.10)^a^	6.15 (2.62-14.40)^a^
Single parent	4.56 (2.92-7.12)^a^	3.66 (2.31-5.78)^a^	5.01 (2.70-9.34)^a^	2.40 (1.46-3.95)^a^	1.54 (0.87-2.74)	3.03 (1.68-5.50)^a^
Geographic location						
Rural	1 [Reference]	1 [Reference]	1 [Reference]	1 [Reference]	1 [Reference]	1 [Reference]
Urban	1.07 (0.70-1.62)	1.28 (0.84-1.95)	1.12 (0.62-2.00)	0.66 (0.40-1.10)	0.71 (0.44-1.14)	0.67 (0.38-1.16)
Parental employment						
Employed full or part time	1 [Reference]	1 [Reference]	1 [Reference]	1 [Reference]	1 [Reference]	1 [Reference]
Unemployed	0.80 (0.55-1.18)	0.75 (0.51-1.11)	0.68 (0.41-1.12)	0.63 (0.40-1.09)	0.82 (0.45-1.49)	0.52 (0.26-1.04)

Abbreviation: ACE, adverse childhood experience.

^a^ *P* < .001.

^b^ *P* < .01.

^c^ *P* < .05.

^d^ Other racial category includes Alaskan Native, American Indian, Asian, or mixed race.

## Discussion

In this cross-sectional study combining findings from 3 US national surveys, the results indicated that a large portion of children with a high number of adverse experiences (ie, high ACEs) or a high level of distress symptoms (ie, high symptoms) were not in contact with clinical behavioral services. The untreated portion of the high-risk juveniles was generally between 41% and 63%.

The group receiving the least services was young Black children who had significantly lower levels of clinical contact compared with non-Hispanic White children whether they were high in ACEs or high in ACEs and distress symptoms. Older Hispanic children had low contact compared with non-Hispanic White children for the ACEs and the combined groups. Interestingly, high-risk children and youth in nontraditional family structures were substantially more likely to have clinical contact than their counterparts living with 2 biological parents. It may be that parental divorce and the transition to blended families represent highly disruptive changes or worrisome conditions for caregivers that are likely to trigger clinical contact.

This study confirms what other studies have shown: that when a general population of children is screened for treatable life course and mental health conditions that are known to lead to poor outcomes, nearly half or more of high-risk children and youth do not appear to be getting professional help. Merikangas et al^4^ found only 50% of children aged 8 to 15 years whom the survey diagnosed as having ADHD, conduct disorder, anxiety, or a mood disorder (in the past year) received treatment. Costello et al^3^ found that 55% of adolescents aged 13 to 17 years with a psychiatric disorder did not receive treatment in the past year. This study’s findings, like earlier findings, add weight to the idea that more children and perhaps all children should be screened for the presence of treatable problems. However, this study adds a dimension to previous studies by showing a failure to bring into clinical view not just children with symptoms but those with a high number of childhood adversities as well.

One of the questions being posed by advocates for increased services is whether additional screening should be for ACEs exposure, distress symptoms, or a combination.^25,26,27^ These 2 groups of children—those flagged by a high level of ACEs and those flagged by high levels of symptoms—are not fully overlapping, as can be seen in this study’s findings. Half or more of the children and youth with high ACEs did not have a high level of symptoms, and half of those with high symptom levels did not have high ACEs.

However, the question about the relative advantages of one set of items, the other, or both for the purposes of universal screening is not readily answerable with the present design or set of findings. An argument for symptom screening is that it provides a more valid indicator that a harmful process is under way, considering that adversities can occur with minimal impact. Another argument is that symptoms are easier to match to treatment options because clinical treatment is typically more organized around symptoms (eg, depression or ADHD) than it is around adversities (having an incarcerated parent or being exposed to a crime scene). The fact that adversities and symptoms do not fully overlap might mean that referral based only on adversities would swamp behavioral health resources with children for whom the treatment target was uncertain. If such advantages exist in selection for clinical contact, this study’s data do not suggest that high symptoms are currently being strongly prioritized over high ACEs in decisions to provide clinical contact. Even if they did, it would not address the question of whether additional screening should rely on adversities or symptoms or some combination. The issues about adversity and symptom screening also must consider development stage, given that the appearance of some symptoms following adversities may take time to emerge.

Moreover, in discussions about screening for risk, planners need to recognize that awareness of risk will not automatically lead to more clinical attention. Screening by itself is not enough. Rather, the problem may lie in features such as the inability to access services or resistance to engaging with them.^28^ If these are the problems, then screening may not be an adequate solution. This possibility is highlighted by the comparatively low level of service receipt among younger Black children with higher risk vs White children and among some risk groups with parents with less education compared with those with college-educated parents. A reason for those lower levels of contact may be lack of convenient or affordable access to clinical services or cultural perceptions that promote skepticism about their relevance or value.^29^ Systemic racism and associated disadvantages may create numerous barriers to receipt among Black children. These may include primary care practitioners who do not recognize their symptoms as serious, their family’s prior negative experience with services, a history of experiencing services and/or health care professionals as untrustworthy, and a lack of resources at the particular schools they attend.

The system that provides behavioral health services to children is currently experiencing many pressures and challenges. There are indications that services are scarce in many areas and inadequate to the demand in most.^16,30^ There are critiques that not enough of the professionals who staff these services are trained in evidence-based treatments.^31^ There is concern that many children and families referred to the system never get seen. There are debates about whether more of these screenings and services should be organized through schools and medical settings.^27,32^

Many of these challenges are integral and antecedent to questions about how to screen for additional need. Before increasing referral, practitioners need to be confident that resources exist, that they are equipped with treatments that are effective, and that they have the tools to eliminate obstacles that interfere with treatment delivery and completion.

### Limitations

This study has the benefit of a large and nationally representative sample and an extensive inventory of adversities and symptoms. However, it has several limitations that need to be considered. The cutoffs for determining need were not based on formal service need assessment. They were based on previous studies associating high levels of ACEs and distress symptoms with other indicators of need. Also, the measure of service receipt may not be comprehensive. Services can be conceptualized in a number of ways, but the survey questions may not have encompassed all the ways that would prompt accurate response from interviews. Similarly, the measurement of symptoms in this study does not include all the possible mental health and behavioral problems that might warrant treatment, so it may underestimate the scope of high-risk children and youth. The data in this analysis amalgamated surveys conducted from 2008 to 2014, and the environment may have changed in more recent years, with some increased vigilance around ACEs, such that the findings do not represent current conditions.

## Conclusions

This study found that many high-risk children and youth with serious behavioral symptoms and conditions known to lead to poor health outcomes were not receiving services that could help reduce their risk. Those children can be identified through adversities or through mental health symptoms, but these 2 approaches highlight distinctly different populations with only partial overlap. How these assessments can be melded to help high-risk children most effectively is a question that should animate future research in this area.

## References

[1] Friedman RA. Uncovering an epidemic—screening for mental illness in teens. N Engl J Med. 2006;355(26):2717-2719. doi:10.1056/NEJMp068262 17192534

[2] Marie-Mitchell A, O’Connor TG. Adverse childhood experiences: translating knowledge into identification of children at risk for poor outcomes. Acad Pediatr. 2013;13(1):14-19. doi:10.1016/j.acap.2012.10.006 23312855

[3] Costello EJ, He JP, Sampson NA, Kessler RC, Merikangas KR. Services for adolescents with psychiatric disorders: 12-month data from the National Comorbidity Survey-Adolescent. Psychiatr Serv. 2014;65(3):359-366. doi:10.1176/appi.ps.201100518 24233052 PMC4123755

[4] Merikangas KR, He J-P, Brody D, Fisher PW, Bourdon K, Koretz DS. Prevalence and treatment of mental disorders among US children in the 2001-2004 NHANES. Pediatrics. 2010;125(1):75-81. doi:10.1542/peds.2008-2598 20008426 PMC2938794

[5] Merikangas KR, He JP, Burstein M, . Service utilization for lifetime mental disorders in U.S. adolescents: results of the National Comorbidity Survey-Adolescent Supplement (NCS-A). J Am Acad Child Adolesc Psychiatry. 2011;50(1):32-45. doi:10.1016/j.jaac.2010.10.006 21156268 PMC4408275

[6] Prochaska JD, Le VD, Baillargeon J, Temple JR. Utilization of professional mental health services related to population-level screening for anxiety, depression, and post-traumatic stress disorder among public high school students. Community Ment Health J. 2016;52(6):691-700. doi:10.1007/s10597-015-9968-z 26733335 PMC4930415

[7] US Preventive Services Task Force. Screening and treatment for major depressive disorder in children and adolescents: US Preventive Services Task Force recommendation statement. Pediatrics. 2009;123(4):1223-1228. doi:10.1542/peds.2008-2381 19336383

[8] Husky MM, Sheridan M, McGuire L, Olfson M. Mental health screening and follow-up care in public high schools. J Am Acad Child Adolesc Psychiatry. 2011;50(9):881-891. doi:10.1016/j.jaac.2011.05.013 21871370

[9] US Centers for Disease Control and Prevention. Preventing adverse childhood experiences. Reviewed April 3, 2020. Accessed February 9, 2021. https://www.cdc.gov/violenceprevention/childabuseandneglect/aces/fastfact.html

[10] Felitti VJ, Anda RF, Nordenberg D, . Relationship of childhood abuse and household dysfunction to many of the leading causes of death in adults: the Adverse Childhood Experiences (ACE) Study. Am J Prev Med. 1998;14(4):245-258. doi:10.1016/S0749-3797(98)00017-8 9635069

[11] Nurius PS, Fleming CM, Brindle E. Life course pathways from adverse childhood experiences to adult physical health: a structural equation model. J Aging Health. 2019;31(2):211-230. doi:10.1177/0898264317726448 28845729 PMC12164666

[12] Shonkoff JP, Garner AS; Committee on Psychosocial Aspects of Child and Family Health; Committee on Early Childhood, Adoption, and Dependent Care; Section on Developmental and Behavioral Pediatrics. The lifelong effects of early childhood adversity and toxic stress. Pediatrics. 2012;129(1):e232-e246. doi:10.1542/peds.2011-2663 22201156

[13] Turner HA, Finkelhor D, Mitchell KJ, Jones LM, Henly M. Strengthening the predictive power of screening for adverse childhood experiences (ACEs) in younger and older children. Child Abuse Negl. 2020;107:104522. doi:10.1016/j.chiabu.2020.104522 32731172

[14] Stavely Z. California wants to find out if you—or your kids—have experienced trauma. Published June 25, 2019. Accessed February 9, 2021. https://edsource.org/2019/california-wants-to-find-out-if-you-or-your-kids-have-experienced-trauma/614217

[15] Choi KR, McCreary M, Ford JD, Rahmanian Koushkaki S, Kenan KN, Zima BT. Validation of the traumatic events screening inventory for ACEs. Pediatrics. 2019;143(4):e20182546. doi:10.1542/peds.2018-2546 30837293

[16] Marrast L, Himmelstein DU, Woolhandler S. Racial and ethnic disparities in mental health care for children and young adults: a national study. Int J Health Serv. 2016;46(4):810-824. doi:10.1177/0020731416662736 27520100

[17] American Association for Public Opinion Research. Standard Definitions: Final Dispositions of Case Codes and Outcome Rates for Surveys. 9th Edition. AAPOR; 2016.

[18] Kennedy C, Hartig H. Response rates in telephone surveys have resumed their decline. Pew Research Center. Published February 27, 2019. Accessed February 9, 2021. https://www.pewresearch.org/fact-tank/2019/02/27/response-rates-in-telephone-surveys-have-resumed-their-decline/

[19] Czajka JL, Beyler A. Declining Response Rates in Federal Surveys: Trends and Implications. Mathematica Policy Research;2016. Accessed February 9, 2021. https://www.mathematica.org/our-publications-and-findings/publications/declining-response-rates-in-federal-surveys-trends-and-implications-background-paper

[20] Finkelhor D, Turner H, Ormrod R, Hamby SL. Violence, abuse, and crime exposure in a national sample of children and youth. Pediatrics. 2009;124(5):1411-1423. doi:10.1542/peds.2009-0467 19805459

[21] Finkelhor D, Turner HA, Shattuck A, Hamby SL. Violence, crime, and abuse exposure in a national sample of children and youth: an update. JAMA Pediatr. 2013;167(7):614-621. doi:10.1001/jamapediatrics.2013.42 23700186

[22] Finkelhor D, Turner HA, Shattuck A, Hamby SL. Prevalence of childhood exposure to violence, crime, and abuse: results from the National Survey of Children’s Exposure to Violence. JAMA Pediatr. 2015;169(8):746-754. doi:10.1001/jamapediatrics.2015.0676 26121291

[23] Briere J. Trauma Symptoms Checklist for Children (TSCC): Professional Manual. Psychological Assessment Resources; 1996.

[24] Briere J, Johnson K, Bissada A, . The Trauma Symptom Checklist for Young Children (TSCYC): reliability and association with abuse exposure in a multi-site study. Child Abuse Negl. 2001;25(8):1001-1014. doi:10.1016/S0145-2134(01)00253-8 11601594

[25] Finkelhor D. Screening for adverse childhood experiences (ACEs): cautions and suggestions. Child Abuse Negl. 2018;85:174-179. doi:10.1016/j.chiabu.2017.07.016 28784309

[26] ACEs Aware. ACE screening clinical workflows, ACEs and toxic stress risk assessment algorithm, and ACE-associated health conditions: for pediatrics and adults. Published April 2020. Accessed February 9, 2021. https://www.acesaware.org/wp-content/uploads/2019/12/ACE-Clinical-Workflows-Algorithms-and-ACE-Associated-Health-Conditions.pdf

[27] Koita K, Long D, Hessler D, . Development and implementation of a pediatric adverse childhood experiences (ACEs) and other determinants of health questionnaire in the pediatric medical home: a pilot study. PLoS One. 2018;13(12):e0208088. doi:10.1371/journal.pone.0208088 30540843 PMC6291095

[28] Bringewatt EH, Gershoff ET. Falling through the cracks: gaps and barriers in the mental health system for America's disadvantaged children. Child Youth Services Rev. 2010;32(10):1291-1299. doi:10.1016/j.childyouth.2010.04.021 PMC8372847 34413557

[29] Planey AM, Smith SM, Moore S, Walker TD. Barriers and facilitators to mental health help-seeking among African American youth and their families: a systematic review study. Children and Youth Services Review. 2019;101:190-200. doi:10.1016/j.childyouth.2019.04.001

[30] Whitney DG, Peterson MD. US national and state-level prevalence of mental health disorders and disparities of mental health care use in children. JAMA Pediatr. 2019;173(4):389-391. doi:10.1001/jamapediatrics.2018.5399 30742204 PMC6450272

[31] Bruns EJ, Kerns SE, Pullmann MD, Hensley SW, Lutterman T, Hoagwood KE. Research, data, and evidence-based treatment use in state behavioral health systems, 2001–2012. Psychiatr Serv. 2016;67(5):496-503. doi:10.1176/appi.ps.201500014 26695495 PMC5107263

[32] Soneson E, Howarth E, Ford T, . Feasibility of school-based identification of children and adolescents experiencing, or at-risk of developing, mental health difficulties: a systematic review. Prev Sci. 2020;21(5):581-603. doi:10.1007/s11121-020-01095-6 32062764 PMC7305254

